# Pulse Oximetry—A Perioperative Perspective

**DOI:** 10.3390/diagnostics16121812

**Published:** 2026-06-12

**Authors:** Kellie Moon, Naema Daino, Paula Gomez, Juan Arias, Ammar Toubasi, Sri Varsha Pulijal

**Affiliations:** 1 Medical College of Georgia, Augusta University, 1120 15th Street, Augusta, GA 30912, USA; kemoon@augusta.edu (K.M.); pgomezparra@augusta.edu (P.G.); jariasbolanos@augusta.edu (J.A.); atoubasi@augusta.edu (A.T.); 2 Department of Anesthesiology and Perioperative Medicine, Augusta University, 1120 15th Street, Augusta, GA 30912, USA; ndaino@augusta.edu

**Keywords:** pulse oximetry principles, photoplethysmography, hemoglobin oxygen saturation monitoring, pulse oximetry interference, perioperative monitoring and clinical applications, pleth variability index

## Abstract

Pulse oximetry is an essential standard monitor in modern anesthetic practice, enabling continuous noninvasive assessment of arterial oxygen saturation and pulse rate throughout the perioperative period. Since its introduction into clinical medicine, pulse oximetry has significantly improved patient safety by facilitating early detection of hypoxemia and physiologic deterioration. Despite its widespread use, clinicians may underrecognize the technical principles, physiologic assumptions, and limitations that influence measurement accuracy. This review provides a perioperative perspective on pulse oximetry, including the physics of photoplethysmography, sensor technologies, and practical considerations for optimal probe placement and signal acquisition. Sources of inaccuracy such as motion artifact, low perfusion states, dyshemoglobinemias, ambient light interference, skin pigmentation, and venous pulsation are discussed in detail. The review further examines perioperative applications across preoperative evaluation, intraoperative monitoring, and postoperative recovery, while also exploring advanced parameters including perfusion index (PI) and pleth variability index (PVI). Emerging innovations such as multi-wavelength systems and artificial intelligence (AI)-enhanced signal analysis are also highlighted. A comprehensive understanding of pulse oximetry allows anesthesiologists to appropriately interpret monitor data, recognize device limitations, and optimize perioperative patient care.

## 1. Introduction

Pulse oximetry is among the most important advances in perioperative monitoring and has become indispensable in anesthetic practice worldwide. Before its widespread adoption in the 1980s, detection of hypoxemia often relied on intermittent arterial blood gas analysis or recognition of late clinical signs such as cyanosis, both of which lacked sensitivity and timeliness. The introduction of pulse oximetry transformed perioperative safety by providing continuous, real-time, noninvasive monitoring of arterial oxygen saturation, enabling earlier recognition of respiratory compromise and reducing anesthesia-related morbidity and mortality.

Today, pulse oximetry is considered a standard of care, with the American Society of Anesthesiologists mandating continuous monitoring of oxygenation during anesthesia [1]. Beyond the operating room, its use now extends to intensive care units, procedural suites, emergency medicine, ambulatory monitoring, and home healthcare settings. Pulse oximeters estimate arterial oxygen saturation (SpO_2_) using spectrophotometric principles and photoplethysmography by analyzing pulsatile changes in light absorption between oxygenated and deoxygenated hemoglobin.

Although pulse oximetry is routinely used by anesthesiologists and perioperative clinicians, interpretation of its readings is not always straightforward. Device accuracy may be influenced by numerous physiologic, technical, and patient-related factors including low perfusion states, motion artifact, dyshemoglobinemias, anemia, skin pigmentation, ambient light exposure, and abnormal venous pulsation. In addition, conventional two-wavelength pulse oximeters provide limited information regarding ventilation, oxygen delivery, and tissue oxygen utilization, which may lead to overreliance on oxygen saturation values alone.

Technological advances over the last decade have expanded the role of pulse oximetry beyond simple oxygen saturation monitoring. Newer systems incorporating reflectance technology, multi-wavelength sensors, signal extraction algorithms, and advanced waveform analysis now allow assessment of perfusion index, pleth variability index, and other hemodynamic parameters that may aid perioperative decision-making.

This review aims to provide a comprehensive perioperative perspective on pulse oximetry by examining its underlying principles, probe technologies, waveform interpretation, clinical applications, limitations, and emerging innovations relevant to contemporary anesthetic practice.

## 2. History

Pulse oximetry builds on nineteenth-century optical principles including the Beer–Lambert law (1852) and Hoppe-Seyler’s hemoglobin studies (1860s). Glenn Millikan’s 1940s work expanding Karl Matthes’ ear oximeter demonstrated light-based oxygenation measurement, but early devices could not separate pulsatile arterial from static tissue signals [2]. Takuo Aoyagi’s 1972 breakthrough of isolating the arterial pulse component enabled continuous noninvasive SpO_2_ monitoring [2]. Following Susumu Nakajima’s 1975 prototype evaluation, Minolta, Biox (Ohmeda), and Nellcor commercialized devices in the late 1970s and early 1980s [3]. Anesthesiologists rapidly adopted the technology for hypoxemia detection, leading to American Society of Anesthesiologists standard incorporation in 1986 and near-universal healthcare adoption by the early 1990s through improved technology and clinical evidence [4].

## 3. Principles of Pulse Oximetry

**Figure 1:**
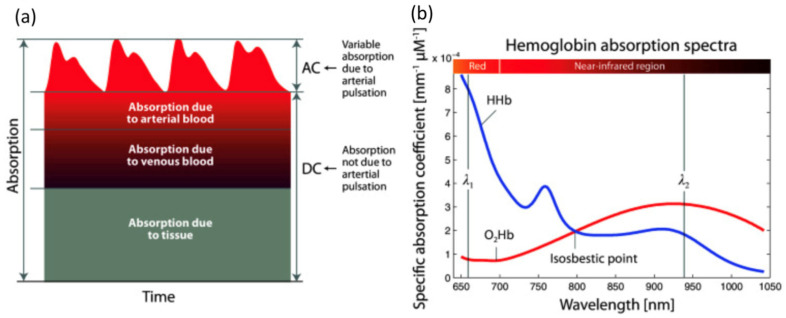
(**a**) Light absorption in various compartments. (**b**) Hemoglobin absorption spectra. ***Reproduced from*** Quaresima V et al. ***J Biomed Opt***. 2024; 29 (Suppl 3): S33307, with permission [4].

**Figure 2:**
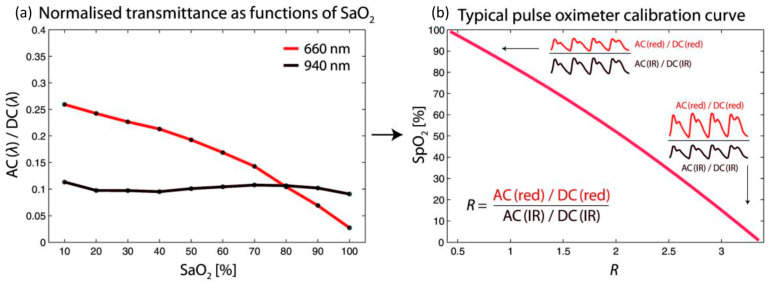
(**a**) Normalized transmittance for two wavelengths as functions of SaO_2_. (**b**) Typical pulse oximeter calibration curve with showing blood volume pulsation in two wavelength ranges. ***Reproduced from*** Quaresima V et al. ***J Biomed Opt*****.** 2024; 29 (Suppl 3): S33307, with permission [4].

Pulse oximetry estimates arterial oxygen saturation (SpO_2_) by analyzing how pulsatile blood absorbs light at two wavelengths [5]. It captures the differential light absorption between oxyhemoglobin (HbO_2_), which preferentially absorbs infrared light (940 nm), and deoxyhemoglobin (Hb), which preferentially absorbs red light (660 nm) (Equation (1) and Figure 1). The probe has two light-emitting diodes delivering these wavelengths and a photodiode detector measuring transmitted light intensity [6]. The physiologic signal underlying pulse oximetry is the photoplethysmographic waveform. By analyzing pulsatile arterial blood flow, the device isolates the arterial signal from static venous and capillary contributions, sampling multiple times per second to separate the pulsatile component (AC signal), generated from the cyclic changes in arterial blood volume, from the nonpulsatile baseline (DC signal), produced by venous blood, capillary blood, skin, bone, connective tissue, and the optical properties of the probe–tissue interface. The ratio of normalized red to infrared absorption expressed as (AC_660_/DC_660_)/(AC_940_/DC_940_) reflects the relative proportions of deoxygenated and oxygenated hemoglobin and is converted to SpO_2_ through calibration algorithms (Figure 2) [7].

(1) $$R=\frac{\left(AC_{660}/DC_{660}\right)}{\left(AC_{940}/DC_{940}\right)}$$

AC is the pulsatile component of the signal. DC is the nonpulsatile component. 660 nm = red light. 940 nm = infrared light.

The Beer–Lambert law provides the theoretical foundation for pulse oximetry. This law states that the concentration of an absorbing substance in solution can be determined from transmitted light intensity, given the incident light characteristics, path length, and substance-specific absorbance at a particular wavelength [6,7]. Although the law provides the theoretical framework for relating light absorption to solute concentration, pulse oximetry is not a direct application of it. Human tissue scatters light, which makes the optical path length variable rather than fixed [8]. Direct application of the Beer–Lambert law significantly overestimates oxygen saturation due to the light being scattered, reflected and absorbed by multiples tissues before reaching the detector [7,8]. For this reason, the relationship between the ratio of ratios and true arterial saturation cannot be derived purely from these principles.

To overcome this limitation, modern pulse oximeters use empirical calibration curves and algorithms derived from healthy volunteers undergoing controlled desaturation, and these calibration datasets are used to estimate functional arterial oxygen saturation from the optical signal [9]. The relationship between the absorption ratio (R) and SpO_2_ is expressed as:

$${SpO}_{2}=a_{0}+a_{1}R+a_{2}R^{2}+\cdots$$

where a_0_, a_1_, and a_2_ are device-specific calibration constants derived by comparing measured R values to arterial blood gas oxygen saturation in healthy volunteers subjected to controlled hypoxia [7]. These calibration curves compensate for the optical properties of tissue and provide clinically accurate saturation estimates.

The same pulsatile signal also enables pulse rate measurement. The photodetector captures the frequency of arterial pulsations, with each pulsation corresponding to a cardiac cycle, and the device counts these to display pulse rate in beats per minute.

A=εcl

Beer–Lambert Law. A = Absorbance. ε = molar absorption coefficient (M^−1^ cm^−1^). c = molar concentration (M). l = optical path length (cm).

## 4. Probes

Pulse oximeter probes can be classified either as transmission or reflectance sensors. Transmission probes place the light source and detector on opposite sides of a tissue with relatively thin vascular beds (commonly finger, toe, or earlobe), allowing light to pass through. Reflectance probes place both components on the same surface and measure reflected light, enabling monitoring at non-transparent sites like the forehead, nasal septum, or chest [10,11]. Probe selection has important clinical implications because signal quality depends not only on the device but also on local perfusion, tissue thickness, temperature, patient movement, surgical positioning, and accessibility of the monitoring site. Understanding each probe’s limitations is essential for accurate readings (Table 1).

## 5. Transmission Probes

Finger probes are standard for routine monitoring. They remain the most widely used sensors because they are inexpensive, easy to apply, and generally accurate in stable patients with adequate peripheral perfusion. However, they may give delayed or false readings during poor circulation, shock, or cold exposure due to reduced blood flow [11,12]. The earlobe is an alternative transmission site because it is closer to central circulation and is usually less affected by peripheral vasoconstriction than the finger. Earlobe probes respond faster to abrupt changes in oxygen saturation and work better in cold conditions. In a clinical trial of 67 patients admitted to the intensive care unit (ICU) after coronary artery bypass surgery, Seifi et al. compared finger, toe, forehead, and earlobe probes against arterial oxygen saturation measured by arterial blood gas (ABG) analysis and found that the earlobe probe showed the best overall accuracy, with the highest correlation and clinical agreement with SaO_2_ and the lowest mean difference from the arterial reference (0.14 ± 0.86%), whereas the forehead probe showed the poorest agreement. These findings suggest that the earlobe may be a more reliable transmission site when peripheral perfusion is compromised, although accuracy still depends on the clinical setting and patient condition [13]. Despite these advantages, earlobe probes are more prone to displacement during surgery, which limits their intraoperative practicality [10], and pressure-related skin injury remains a concern during prolonged use [8].

## 6. Reflectance Probes

Reflectance probes represent a clear advantage, especially when peripheral perfusion is compromised or when hands are inaccessible. Forehead sensors are the most used reflectance probes in anesthetic and perioperative practice. Due to being more centrally perfused than the fingers, they often maintain a stable signal during vasoconstriction, hypothermia, and low-flow states, and may detect oxygenation changes earlier than finger probes. Robertson et al. conducted a study that compared forehead and finger oximetry in 80 patients with pulmonary vascular disease and/or interstitial lung disease during the six-minute walk test (6MWT). The forehead sensor had significantly fewer poor-quality signals than the finger sensor (34/1040 vs. 189/1040, *p* < 0.001), supporting the potential signal stability advantage of the forehead positioning during motion. However, the measurement site also had a significant impact on the values obtained, with the forehead sensor recording higher SpO_2_ and heart rate values than the finger sensor. Importantly, although the forehead probe was more reliable in terms of signal quality, it tended to overestimate oxygen saturation compared with capillary oxygen saturation (ScO_2_), while the finger sensor showed less bias but greater variability in agreement with ScO_2_. Notably, both sensors appeared to have limited accuracy in reflecting ScO_2_, particularly in patients who desaturated during exercise. These findings underscore that pulse oximetry may not fully capture the degree of exercise-induced desaturation and suggest that blood gas measurements should be considered alongside oximetry when important clinical decisions are being made, such as ambulatory oxygen prescription [14].

While generally stable during patient repositioning, forehead probes may give falsely low readings from venous blood pulsations, especially in Trendelenburg positioning, elevated venous pressure, or when applied too tightly. In practice, using a tensioned elastic headband may reduce this issue [10], although current evidence more strongly supports improved signal conditions than a direct causal claim that the headband itself eliminates measurement error [14].

No single probe site is ideal for all patients or surgical settings. Rather than viewing alternative probes as interchangeable, clinicians should select the site most likely to provide the most reliable signal under the patient’s physiologic conditions. In routine, well-perfused patients, a finger probe is usually sufficient. In low-perfusion states, during procedures involving the hands, or when rapid desaturation detection is important, earlobe, forehead, or nasal sites may offer practical advantages and may reduce signal degradation associated with peripheral vasoconstriction or motion artifact [15].

## 7. Limitations and Sources of Error

Pulse oximetry accuracy is affected by skin pigmentation, dyshemoglobinemias, temperature, hypotension, perfusion status, motion artifact, and optical interference through several distinct physiologic and technical mechanisms [6] (Table 2). When the readings are discordant with the clinical picture assess probe contact, site, and positioning first. Evaluate perfusion and waveform quality, consider patient-specific confounders (pigmentation, dyshemoglobinemias, motion) and confirm with ABG or co-oximetry when uncertainty persists.

## 8. Optical Interference

Conventional two-wavelength pulse oximeters cannot distinguish oxyhemoglobin from dyshemoglobins. Carboxyhemoglobin (COHb) falsely elevates SpO_2_ due to spectral similarity to oxyhemoglobin; in carbon monoxide poisoning, readings may appear normal despite life-threatening hypoxia, requiring co-oximetry for diagnosis [10]. Methemoglobin (MetHb) impairs oxygen binding, and pulse oximetry often trends toward a reading near 85% regardless of true saturation [6]. In their recommendations on methemoglobinemia, Iolascon et al. emphasized that the diagnosis should be confirmed by co-oximetry, which directly measures MetHb levels. They also noted that even relatively low levels may produce misleading pulse oximeter findings. MetHb < 10% may already be associated with low pulse oximeter readings and skin color changes, while levels between 10% and 30% are associated with cyanosis and dark brown blood [16]. Wakita et al. reported a perioperative case that described a 43-year-old woman who developed methemoglobinemia after prilocaine infiltration during intravenous sedation. In the case, SpO_2_ decreased from 98% at baseline to sustained values between 85% and 87% despite recovery of sedation level and oxygen administration. Venous co-oximetry showed MetHb levels of 10.6% initially and 11.4% later. These findings support that a persistent SpO_2_ near 85%, particularly when discordant with the clinical picture, should prompt consideration of methemoglobinemia and confirmation with co-oximetry [17].

Current U.S. Food and Drug Administration (FDA) guidelines highlight that device performance can vary across clinical and patient factors, including skin pigmentation, perfusion, and motion artifact. Increasing evidence of pulse oximeter variability across skin tones has led to updated FDA recommendations emphasizing more diverse validation cohorts and improved subgroup reporting during device evaluation. The guidance also encourages standardized skin tone classification methods rather than relying solely on self-reported race or ethnicity [18]. One proposed method for standardizing skin tone assessment is the Monk Skin Tone (MST) scale, a 10-point visual classification system designed to better represent the diversity of human skin pigmentation. Compared with broad racial or ethnic categories, the MST scale provides a more consistent framework for evaluating device performance across different skin tones and may improve comparability between studies [19]. Darker skin pigmentation may cause overestimation of oxygenation, particularly during hypoxemia [15]. In their review, Al-Halawani et al. found that 22 of 28 studies reported overestimation of oxygen saturation by pulse oximetry in individuals with darker skin. Additionally, 57% of the studies also showed increased bias at lower oxygen saturations, with both bias and precision showing wider ranges in Black subjects compared with White subjects [20]. Similarly, Martin et al. analyzed 44 studies including 222,644 participants and 733,722 paired SpO_2_-SaO_2_ measurements and found that occult hypoxemia was more common in Black patients than in White patients (pooled prevalence ratio of 1.67; 95% confidence interval [CI] = 1.47–1.90) and more common in other racial and ethnic minority groups (pooled prevalence ratio of 1.39; 95% CI = 1.19–1.64). These data support describing skin tone as a clinically relevant limitation of pulse oximetry rather than merely a theoretical source of variability [21].

Optical interference from exogenous substances can also alter measured SpO_2_. Intravenous dyes, especially methylene blue, cause transient false desaturation with SpO_2_ decreases of 10–15% lasting up to 30 min despite stable arterial oxygenation [10]. Dark nail polish, tattoos, severe anemia, ambient light, and rare hemoglobin variants (e.g., Hb Köln, Hb Cheverly) can further distort readings, potentially requiring arterial blood gas confirmation [10,15]. Although many of these effects are modest under ideal conditions, they become more clinically relevant in low-perfusion states, during movement, or when clinicians rely on a single suboptimal monitoring site [22,23,24,25].

## 9. Calibration Limitations

Pulse oximeters use empirically derived calibration curves from healthy volunteers. Most commercial devices are calibrated primarily over saturation ranges of approximately 70% to 100%. Accuracy declines below 80% saturation and becomes unreliable below 70%, limiting utility in severe hypoxemia [6]. Agreement between SpO_2_ and SaO_2_ may additionally vary according to device manufacturer, signal quality, perfusion, motion artifact, and patient-specific characteristics.

Finally, pulse oximetry measures oxygen saturation rather than oxygen content and ventilation. Therefore, a normal or near-normal SpO_2_ does not exclude hypercapnia, hypoventilation, severe anemia, or impaired oxygen delivery. This distinction is particularly important in the perioperative setting, where supplemental oxygen can preserve arterial saturation despite progressive ventilatory failure. For that reason, pulse oximetry should be interpreted in conjunction with the overall clinical context and, when relevant, with capnography, arterial blood gas, or co-oximetry [26].

## 10. Different Waveforms and Interpretations

Pulse oximetry estimates SpO_2_ using the ratio (R) of red to infrared light absorbance during pulsatile flow. When R = 1, the device calculates SpO_2_ as approximately 85%. With poor perfusion, probe displacement, or motion artifact, the algorithm cannot calculate a valid R value and defaults to R ≈ 1, displaying ~85% regardless of true saturation. Clinicians should recognize this as a technical artifact, not a physiologic measurement [8] (Table 3).

In mechanically ventilated patients, respiratory variation in the plethysmographic waveform suggests fluid responsiveness. Positive-pressure inspiration reduces venous return and stroke volume in preload-dependent states, producing significant waveform variation (typically > 15–20%) indicating hypovolemia. Minimal variation suggests adequate volume status or conditions limiting fluid responsiveness: right ventricular dysfunction, high positive end-expiratory pressure (PEEP), spontaneous breathing, or arrhythmias [27].

**Figure 3:**
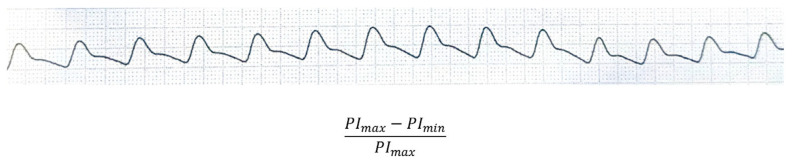
Pulse oximetry plethysmography showing variability and formula to calculate pleth variability index (PVI). PI = perfusion index.

The pleth variability index (PVI), introduced by Masimo Corporation in 2007, automates quantification of respiratory waveform variation using maximum and minimum perfusion indices over the respiratory cycle [27] (Figure 3). In patients with controlled ventilation and regular rhythm, PVI > 13–14% indicates fluid responsiveness and correlates with arterial pulse pressure variation [27]. PVI can also assess pulsus paradoxus [27].

Perfusion index (PI) is additionally clinically useful for measurements that can be used for noninvasive hemodynamic monitoring. PI is calculated as the ratio of alternating current (AC) and direct current (DC) (Equation (2)). The AC component is generated from pulsatile vessels, whereas the DC component reflects non-pulsatile vessels, bone, and soft tissues. PI is affected by both intrinsic and external factors with the main determinants being stroke volume and vascular tone. PI can be used across multiple clinical settings, including during regional and general anesthesia, as well as in the intensive care unit (ICU). During general anesthesia, PI is used to measure PVI, which predicts fluid responsiveness in mechanically ventilated patients [28].

(2) $$Perfusion\;Index=\frac{AC}{DC}$$

Formula to calculate perfusion index (PI). AC = alternating current. DC = direct current.

### Perioperative Period

The World Health Organization (WHO) includes pulse oximetry in its Safe Surgery Checklist as essential for perioperative safety [29]. Pulse oximetry reduces postoperative complications and mortality through rapid hypoxemia detection and real-time intervention feedback [30,31,32]. Despite being standard of care, many healthcare providers have inadequate knowledge of its principles, normal values, and accuracy factors [33].

## 11. Preoperative Assessment

Baseline SpO_2_ informs anesthetic planning and risk stratification. Preoperative SpO_2_ < 95% is associated with increased postoperative respiratory complications and warrants evaluation and optimization [34].

The 6 min walk test with pulse oximetry provides an objective functional assessment. Exercise-induced desaturation (≥4% decline or SpO_2_ < 90%) predicts increased perioperative risk, particularly for lung resection candidates. Cardiopulmonary exercise testing (CPET) is another diagnostic tool to assess cardiorespiratory fitness. This test has been used to assess the risk of cardiovascular diseases among athletes and monitor disease progression [35].

Plethysmographic waveform analysis offers noninvasive fluid responsiveness assessment. While arterial pulse pressure variation remains the gold standard [36], pulse oximetry waveform variability correlates strongly with arterial measurements in critically ill patients [37] and post-cardiac surgery [38]. Respiratory waveform changes also occur in obstructive airway disease, pericardial effusion, and cardiac tamponade, detecting pulsus paradoxus with normalization after pericardiocentesis [39].

## 12. Intraoperative Monitoring

The American Society of Anesthesiologists mandates continuous pulse oximetry with audible alarms during all anesthetics [1]. While anesthesia machines monitor inspired oxygen with low-oxygen alarms, pulse oximetry quantifies actual patient oxygenation.

Variable-pitch auditory signals convey real-time oxygen saturation and pulse rate changes without requiring visual attention. Simulated studies showed anesthesiologists using variable-pitch oximetry detected hypoxemia faster and intervened earlier than with fixed-pitch systems [31], providing critical awareness during multitasking in busy operating rooms.

Maintaining SpO_2_ > 95% during general anesthesia provides safety margin on the oxyhemoglobin dissociation curve (PaO_2_ ~ 80 mmHg at 95% vs. ~60 mmHg at 90%). For chronic respiratory disease patients, targeting SpO_2_ > 88% may be appropriate.

Induction and emergence are highest-risk periods for hypoxemia, commonly caused by atelectasis, apnea, laryngospasm, residual neuromuscular blockade, and hypoventilation [32]. Pulse oximetry reduces hypoxemia incidence and improves ventilation problem detection [30], guiding fraction of inspired oxygen (FiO_2_) adjustments [11].

Acute desaturation suggests circuit disconnection, endobronchial intubation, laryngospasm, aspiration, or pneumothorax, while gradual decline indicates atelectasis or ventilation–perfusion mismatch.

A randomized trial of >20,000 surgical patients showed pulse oximetry increased hypoxemia detection in operating rooms and post-anesthesia care units (PACUs), with lower myocardial ischemia rates [40].

## 13. Postoperative Care

American Society of Anesthesiologists post-anesthesia standards require quantitative oxygenation assessment, routinely achieved with pulse oximetry [41]. Pulse oximetry monitoring increases interventions including oxygen titration and naloxone administration [42], enabling targeted rather than universal supplemental oxygen therapy and potentially reducing costs [43].

Although pulse oximetry has not reduced postoperative complications or hospital stays, 18% of anesthesiologists reported avoiding serious complications and 80% felt greater confidence using it [30,42]. Capnography provides complementary monitoring, detecting adverse respiratory events earlier, and facilitating timely intervention [41].

Future research should investigate whether systematic preoperative oxygen saturation documentation and optimization improve perioperative outcomes and mortality.

## 14. Recent Advances and Innovations

Multi-wavelength pulse oximeters extend beyond the conventional two-wavelength design by incorporating additional light sources that reduce tissue interference, mitigate motion and venous pulsation artifacts, and enable detection of dyshemoglobinemias—including carboxyhemoglobin and methemoglobin—that standard devices cannot identify [3,23]. Masimo’s Rainbow Pulse Co-Oximetry exemplifies this approach, providing noninvasive measurement of hemoglobin concentration, carboxyhemoglobin, methemoglobin, SpO_2_, pulse rate, perfusion index (PI), and pleth variability index (PVI) [23]. Continuous noninvasive detection of dyshemoglobinemias is among the most clinically significant advances enabled by this technology, as it provides information previously obtainable only through laboratory co-oximetry. Accuracy remains susceptible to low-perfusion states and severe vasoconstriction [44].

Traditional hemodynamic parameters—including heart rate, blood pressure, and central venous pressure—poorly predict fluid responsiveness [23]. The pleth variability index (PVI) addresses this gap by quantifying respiratory variation in the plethysmographic waveform; PVI-guided fluid therapy during abdominal surgery has been associated with reduced overall fluid administration and lower postoperative lactate levels [25,45,46]. Compared with invasive dynamic preload indices such as stroke volume variation or pulse pressure variation, PVI offers the advantage of continuous noninvasive monitoring, but its reliability is highly condition-dependent. Optimal performance requires controlled mechanical ventilation with fixed tidal volumes and sinus rhythm; accuracy diminishes during spontaneous ventilation, arrhythmias, vasopressor use, low tidal volume ventilation, or impaired peripheral perfusion. PVI is therefore best regarded as an adjunctive tool for fluid responsiveness assessment rather than a standalone hemodynamic endpoint [47,48].

The perfusion index (PI) complements PVI by providing a quantitative measure of peripheral pulsatile signal strength, expressed as the ratio of pulsatile to non-pulsatile flow (range approximately 0.02% to 20%). A PI above 1% generally indicates adequate signal quality for reliable SpO_2_ measurement [25] and has been investigated as a noninvasive marker of peripheral perfusion and vascular tone in both the operating room and intensive care unit.

Artificial intelligence represents a distinct advance in pulse oximetry, improving signal interpretation through machine learning rather than through additional wavelengths or derived indices [49]. Leading methodologies include deep neural networks for complex photoplethysmography (PPG) waveform analysis, Gaussian process models that characterize signal relationships while incorporating uncertainty, and ensemble methods that combine multiple model outputs to improve SpO_2_ estimation [50,51,52]. In a recent systematic review, Gaussian process models achieved mean absolute errors as low as 0.57% and root mean square errors of 0.69% [50]. Despite improved performance during motion artifact and low-perfusion conditions, most AI-enabled systems remain investigational. Key limitations include training on conventional pulse oximeter outputs rather than arterial co-oximetry reference standards, algorithm opacity, dataset heterogeneity, and reduced accuracy across diverse skin tones—concerns that must be addressed before routine perioperative integration [49,53,54,55].

Wearable technologies—including smartwatches and finger rings—have shifted pulse oximetry from episodic clinical measurement toward continuous ambulatory monitoring using reflective photoplethysmography [56]. In a study of 200 patients, the Apple Watch achieved 84.9% overall accuracy (sensitivity 34.8%, specificity 97.5%) and the Withings ScanWatch 78.5% accuracy (sensitivity 68.5%, specificity 80.8%) for detecting clinically significant hypoxemia [44]. A separate study in a diverse cohort spanning a range of skin tones found root mean square deviation (RMSD) values of 2.6% for smartphone-based and 1.8% for smartwatch-based SpO_2_ estimation [57]. While these results suggest acceptable performance under many ambulatory conditions, wearable devices are more susceptible to motion artifact and perfusion-related signal degradation than conventional transmission probes, and accuracy is further reduced in individuals with darker skin pigmentation [44,57]. In perioperative practice, their role is likely confined to postoperative surveillance and remote monitoring rather than replacement of clinical-grade oximetry in high-risk settings [58].

Collectively, these innovations reflect pulse oximetry’s evolution from a single-parameter saturation monitor to a platform supporting hemodynamic, physiologic, and predictive assessment. Multi-wavelength co-oximetry has achieved the broadest clinical integration; AI-enhanced systems and wearable platforms require further validation—particularly across diverse patient populations—before routine perioperative adoption. Future development should prioritize demonstrating not only technical accuracy but meaningful improvement in clinical outcomes.

## 15. Conclusions

Pulse oximetry has evolved from simple saturation monitoring into a sophisticated multiparameter tool integral to perioperative safety. Contemporary advances like multi-wavelength co-oximetry, pleth variability indices, improved reflectance sensors, and AI-enhanced signal processing demonstrate continued adaptation to clinical needs. As precision monitoring becomes central to modern anesthesia, pulse oximetry remains foundational, complementing arterial blood gas analysis, near-infrared spectroscopy, and other modalities in guiding safe, evidence-based patient management.

## Figures and Tables

**Table 1:** Advantages and disadvantages of pulse oximetry probe sites.

Probe Site	Advantages	Disadvantages
Finger	Most common and easy to apply. Good accuracy with adequate perfusion.	Affected by vasoconstriction, hypothermia, and shock. Motion artifacts are common. Inaccurate with nail polish or acrylic nails.
Toe	Alternative when hands are unavailable.	Poor perfusion in shock or vasoconstriction. Slower response time. Inaccurate with nail polish or acrylic nails
Earlobe	Less affected by peripheral vasoconstriction, faster response to desaturation.	Requires specific probe, pressure injury with prolonged use and prone to displacement intraoperatively.
Forehead (reflectance)	Less affected by peripheral vasoconstriction, faster response to desaturation. Less motion artifacts.	Highest cost, affected by ambient light/sweating. Falsely low readings due to venous pulsations.
Nasal	Centrally perfused site with rapid detection of hypoxemia. Useful in low peripheral flow.	Limited availability, risk of skin injury, interference from secretions.
Tongue	Excellent signal in severe vasoconstriction with rapid response to hypoxemia.	Invasive and uncomfortable, impractical in awake patients, risk of injury and interference from secretions.
Palm or Sole	Useful when digits are unavailable.	Poor signals in thick skin individuals and prone to motion artifacts.
Wrist	Convenient and wearable.	Poor accuracy in low perfusion states and prone to motion artifacts.

**Table 2:** Limitations of pulse oximetry: sources of error and mitigation strategies.

Limitation/Condition	Typical SpO_2_ Effect (Bias)	Clinical Implication	Mitigation /Confirmatory Test
Does not measure ventilation or PaO_2_; cannot detect hyperoxemia; supplemental O_2_ may mask hypoventilation	SpO_2_ may remain “acceptable” despite ventilatory failure; hyperoxemia unrecognized	False reassurance (adequate SpO_2_ ≠ adequate ventilation/PaO_2_)	Add capnography/EtCO_2_ and/or ABG when ventilation or PaO_2_ is clinically relevant
Carboxyhemoglobinemia (COHb)	False high/normal SpO_2_	CO poisoning can be missed with standard 2-wavelength oximetry	Co-oximetry (multi-wavelength) required for diagnosis
Methemoglobinemia (MetHb)	SpO_2_ often “fixed” near 85%	Persistent value of 85% may not reflect true SaO_2_	Confirm with co-oximetry/ABG when suspected
Intravenous dyes (methylene blue, indigo carmine, indocyanine green	Transient false low SpO_2_	Apparent desaturation despite stable arterial oxygenation	Recognize timing after dye administration; avoid reflex escalation if other parameters are stable
Low peripheral perfusion (shock, hypothermia, vasopressors	Under-reading (reported up to 4–6%) or no value	Missed/delayed detection; intermittent or absent signal	Consider forehead reflectance probe; optimize perfusion and evaluate pleth quality
Venous pulsations (forehead probe; Trendelenburg, ↑ CVP, overly tight band)	False low (up to approximately 5% below arterial saturation)	Underestimation may prompt unnecessary intervention	Adjust headband tension and/or change probe site
Motion artifact (shivering, restlessness, seizures)	Unpredictable erroneous values	Spurious desaturation or misleading stability	Secure probe, reduce motion when feasible, corroborate with additional monitoring
Skin pigmentation variability and nail polish (dark colors)	Small bias possible with hyperpigmentation; often false low with dark polish	Discordant SpO_2_ vs. clinical picture	Switch site (earlobe/forehead) if needed; remove polish if possible
Ambient light (bright OR lights, direct sunlight)	Unstable/fluctuating readings	Artifact may mimic physiologic change	Shield sensor with opaque covering; reposition probe
Severe anemia	Possible underestimation, especially during hypoxemia	Under-reading may complicate interpretation in compromised patients	Interpret cautiously; consider ABG if clinical concern persists
Rare hemoglobin variants (e.g., Hb Köln, Hb Cheverly)	False low SpO_2_	Persistent discordance despite adequate oxygenation	Confirm with ABG and evaluate for Hb variant
Calibration/training dependence	Systematic error or misuse	Accuracy depends on calibration curves and correct implementation	Emphasize training and standardized troubleshooting; verify pleth quality
When the pleth signal is compromised, readings may artifactually stabilize near 85% (R ≈ 1; motion/noise/poor perfusion)	SpO_2_ near 85% despite unreliable signal	Can be mistaken for true desaturation	Treat as signal failure: check probe contact, site, perfusion, artifact; corroborate clinically

ABG = arterial blood gas; COHb = carboxyhemoglobin; CVP = central venous pressure; EtCO_2_ = end-tidal carbon dioxide; MetHb = methemoglobin; PaO_2_ = arterial oxygen tension; SpO_2_ = peripheral oxygen saturation.

**Table 3:** Plethysmograph waveforms and interpretations.

Waveforms	Interpretations
** 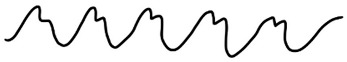 **	Normal signal—Sharp upstroke reflecting good arterial compliance and perfusion; dicrotic notch indicating normal aortic valve closure; regular rhythm with consistent pulse intervals; strong amplitude suggesting adequate stroke volume and peripheral blood flow.
** 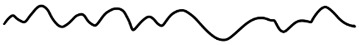 **	Low amplitude—Reduced plethysmographic waveform suggesting decreased pulsatile flow: hypoperfusion, vasoconstriction, hypothermia.
** 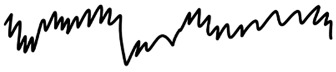 **	Sawtooth or erratic waveform—Rapid, jagged baseline oscillations: shivering, tremors, patient movement.
** 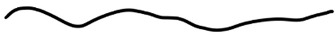 **	Flattened waveform—Low amplitude and loss of normal waveform contour, demonstrating significantly impaired perfusion: hypovolemia, low cardiac output.
** 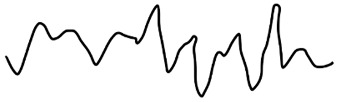 **	Irregular contour—Inconsistent waveforms beat to beat, displaying uneven intervals between pulses with variable amplitudes and no consistent pattern of spacing or size: motion artifact, arrhythmias.

## Data Availability

No new data were created or analyzed in this study.
